# Ulcerated breast cancer with single brain metastasis: A combined surgical approach. Clinical presentation at one year follow up – A case report

**DOI:** 10.1016/j.ijscr.2020.06.074

**Published:** 2020-06-20

**Authors:** Francesca Santori, Gianluca Vanni, Oreste Claudio Buonomo, Adriano De Majo, Maurizio Rho, Alessandra Vittoria Granai, Marco Pellicciaro, Maria Cotesta, Massimo Assogna, Rolando Maria D’Angelillo, Marco Materazzo

**Affiliations:** a Breast Unit, Department of Surgical Science, Policlinico Tor Vergata University, Viale Oxford, 81, 00133, Rome, Italy; b Department of Radiation Oncology, Policlinico Tor Vergata University, Viale Oxford, 81, 00133, Rome, Italy

**Keywords:** BC, breast cancer, BC_IV_, breast cancer de novo stage IV, PTR, primary tumour resection, B-MRI, brain magnetic resonance imaging, PET-CT, positron emission tomography-computerized tomography, BR, breast reconstruction, WBRT, whole brain radiation therapy, EBRT, external beam radiation therapy, SRS, stereotaxis radiosurgery, Breast cancer, Brain metastasis, Breast reconstruction, Metastasectomy, Case report

## Abstract

**Introduction:**

Breast cancer is the most common malignancy in woman. Approximately 5–10% of breast cancer occurs as de novo stage IV and some studies have shown that from 10% to 30% of those patients presents Brain Metastasis.

**Presentation of case:**

In this study, we report a case of solitary brain metastasis of breast cancer in a 63-year-old Italian Caucasian woman with neurological symptoms as first clinical presentation. After the correct diagnosis and multidisciplinary meeting it was decided to simultaneously perform a metastasectomy surgery plus right mastectomy, right axillary dissection and immediate breast reconstruction. In our clinical practice we report a successful combined surgical approach in a stage IV de novo breast cancer patient with single site brain metastasis at one year follow-up.

**Discussion:**

Metastasectomy plus mastectomy provided neurological control of acute complication of metastatic disease and complete breast cancer local control. One-time operation could be the best option when diagnosis of breast cancer is made thanks to the onset of oncological emergency like intracranial hypertension due to single brain metastasis.

**Conclusion:**

Combined surgical approach offers the opportunity to treat two different oncological urgencies, reducing the unnecessary repeated surgical and anesthesiologic trauma.

## Highlights

• Solitary brain metastasis of breast cancer in a patient with neurological symptoms as first presentation is a rare complication.
• Simultaneously perform a metastasectomy surgery plus right mastectomy, right axillary dissection and immediate breast reconstruction is unusual event.
• Successful combined surgical approach in a stage IV de novo breast cancer patient with single site brain metastasis at one year follow-up.
• Combined surgical approach offers the opportunity to treat two different oncological urgencies, reducing the unnecessary repeated surgical and anesthesiologic trauma.

## 1 Introduction

Breast cancer (BC) is the most common malignancy in women, affecting 2.1 million women each year and causing 627,000 deaths in 2018 [1]. Approximately 5–10% of breast cancer occurs as *de novo* stage IV (BC_iv_) and some studies have shown that from 10% to 30% of BC_iv_ presents Brain Metastases (BM) [2,3].

The role of primary tumour resection (PTR) in this subset of patients is still unclear and primary retrospective evidence demonstrated better outcome in patients who underwent surgery [[4], [5], [6], [7], [8]].

Here, we report a case of solitary, brain metastasis of BC in a female patient with neurological symptoms as clinical presentation, the subsequent combined surgical approach and one-year disease control result. This work is reported by following the surgical case report (SCARE) guidelines [9].

## 2 Presentation of case

**Fig. 1:**
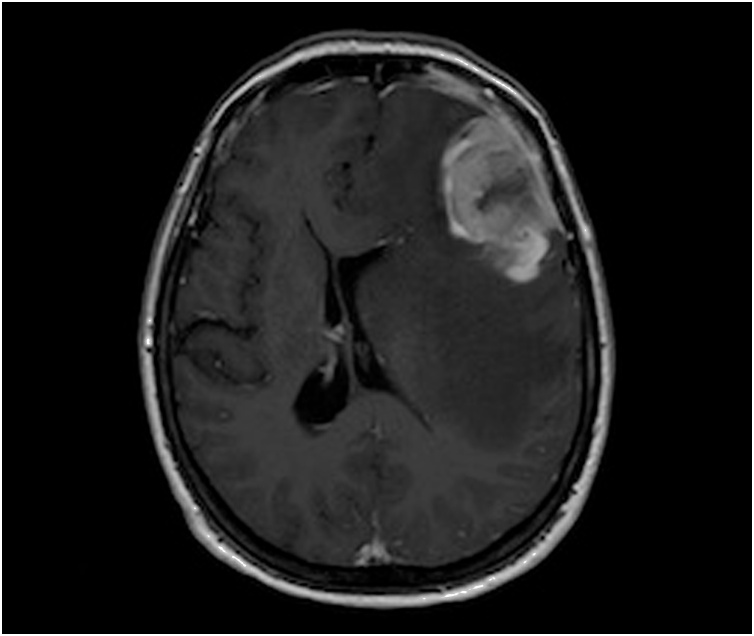
B-MRI shows a single extraxial left frontotemporal lesion of about 52 mm, which determined vasogenic edema and midline 11 mm shift.

**Fig. 2:**
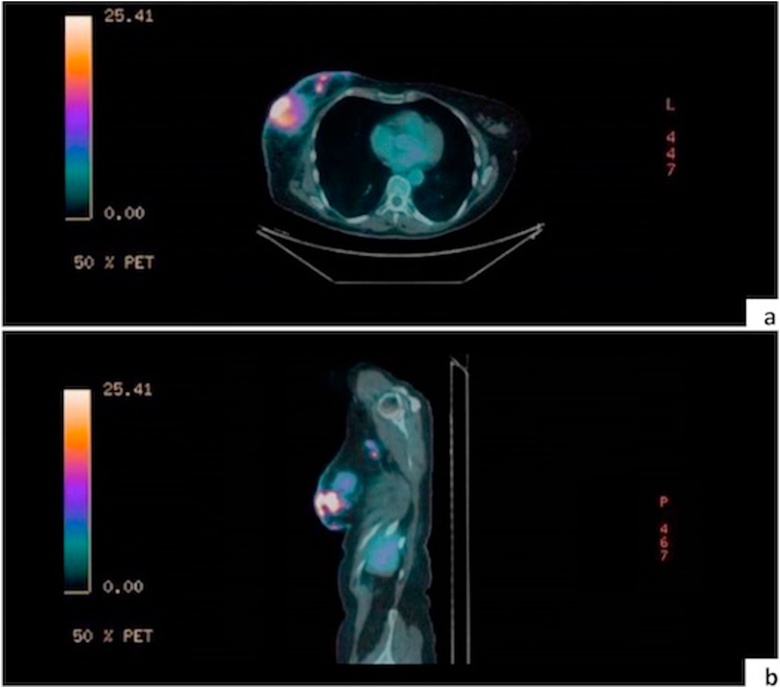
a) Axial PET-CT shows an intense hypermetabolism in right breast. b) Sagittal PET-CT shows an intense hypermetabolism in right breast and right axilla.

**Fig. 3:**
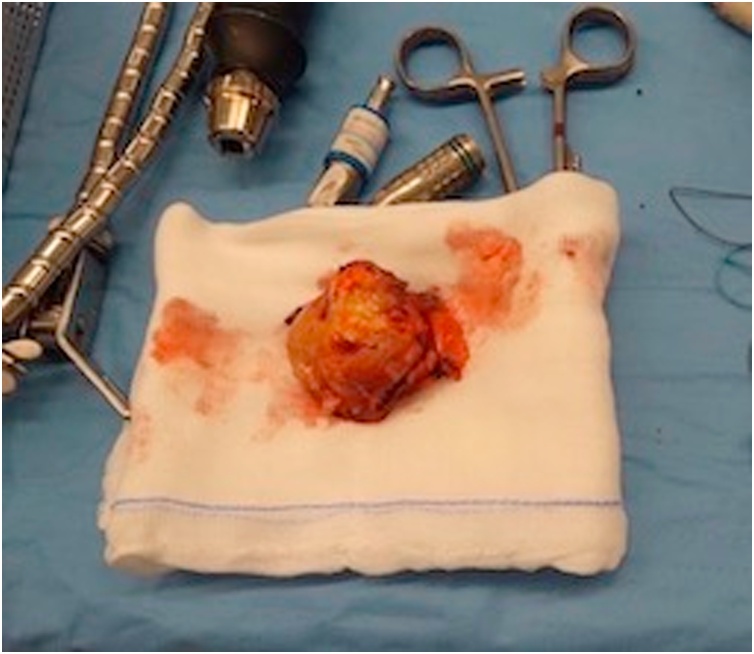
The photo was taken at the end of surgery and shows the brain metastasis of about 52 mm.

A 63-year-old Italian Caucasian woman was admitted in the Emergency Department for right acute arm weakness and one month worsening cognitive motor slowing and aphasia. Family history was negative for cardiovascular and oncological diseases and patient, former smoker of 3,75 pack years, didn’t have any comorbidities. Neurological examination showed amimic expression, aphasia, right reflex hypererexcitabity, unsteady walk and right arm weakness. Thorax examination exhibited a firm deep fixed ulcerated 50 mm breast mass and right lymphadenopathy. Brain Magnetic Resonance Imaging (B-MRI) described a single extraxial left frontotemporal lesion of about 52 mm, which showed contrast enhancement. Mass effect determined vasogenic edema and left hemicortical 11 mm shift (Fig. 1, Fig. 2). After hospitalization intravenous Betamethasone and Furosemide b.i.d. was administrated with partial resolution of aphasia and resolution of motor defect. Positron Emission Tomography - Computed Tomography (PET-CT) has revealed an intense hypermetabolism in right breast and right axilla (SUV max 20,5, 10.5 respectively) (Fig. 2a and Fig. 2b). Core needle biopsy of the breast revealed a grade 3 invasive ductal carcinoma. Immunohistochemistry described estrogen receptor (ER), progesterone receptor (PR) and Ki67 index as 50%, 5% and 20%, respectively. Human epidermal growth factor receptor 2 (HER2) overexpression score was 3+ according to ASCO/CAP 2018 updated guidelines. After pathology, multidisciplinary assessment took place between to Breast Cancer Center Specialist (Breast Surgeon, Medical Oncologist, Breast Radiologist) and Neurosurgeon to address correct clinical management. Multidisciplinary meeting decided for combined surgery upfront in order to treat clinical emergencies (Ulcerated Breast Neoplasm, Endocranic Hypertension) instead of primary medical treatment. After written informed consent, patient underwent same time metastasectomy surgery (Fig. 3) plus right mastectomy, right axillary dissection and immediate Breast Reconstruction (BR) with sub-pectoral Mentor 450 cc tissue expander. Lymphadenectomy was performed due to the clinical evidence of lymphadenopathy. Post-operative course was regular, patient exhibited slight frontal lobe syndrome and complete resolution of clinical neurological presentation. Patient was fully mobilized in second postoperative day and discharged on sixth post-operative day. Final histological examination of specimens confirmed the presence of metastatic multifocal infiltrating ductal carcinoma of the breast. Pathological axillary specimen confirmed PET-CT findings with 8 out 20 metastatic lymph node (pT4b, N2, M1). Subsequent CT with contrast enhancement was performed and no further distant disease was confirmed (R0). After surgical recovery, Whole Brain Radiation Therapy (WBRT) plus Stereotaxis boost on the surgical site was administered followed by Paclitaxel-Pertuzumab-Trastuzumab scheme plus breast External Beam Radiation Therapy (EBRT). During follow up patient returned without symptoms of new onset and no evidence of disease at 1 year. Patient is still now under strict follow up and will be send soon to Plastic Surgeon facility to plan her breast reconstruction.

## 3 Discussion

BM represents the most common intracranial tumors in adults and breast cancer is the second most common cause [10,11]. In most of the cases BM is a late distant recurrence site after bones, liver, lung or skin and only 17% experienced BM as first site of metastasis. BC BM typically occurs between 2 and 3 years after diagnosis, while some studies demonstrated synchronous BM (within 1 month) occurs in almost one third of patients [12].

BM rate risk is usually highest for women with more aggressive molecular subtypes, such as HER2-positive or triple-negative BC [13]. In particular, HER2 type is the most common associated with BM with an incidence ranging from 30% to 53% [14]. BC_iv_ prognosis remains poor, despite survival rate patients is improved in the last 30 years [15]. The role of PTR in *de novo* BC_iv_ is still under investigation, but evidences in literature show how multidisciplinary protocol (systemic treatment plus PTR) could provide better outcome if no evidence of residual disease is achieved, especially in HER2 type patients [4,16].

BM symptoms includes headache (50%), focal weakness (40%), confusion or altered mental status (30%), seizures (15%), ataxia (10%). Moreover, diagnosis commonly occurs in emergency situations due to intracranial hypertension like in our clinical case. BM symptoms are likely due to mass effect of the tumor growth and surrounding edema on nearby structures, as underlined by partial regression after intravenous therapy in our patient [12]. Diagnosis of BM is usually made after the appearance of abovementioned symptoms and B-MRI represents the gold standard of treatment. B-MRI easily detects the site, number, size and intrametastatic bleeding; subsequent total body contrast enhancement CT or PET/CT is recommended to highlight primitive tumor site if unknown [12,17]. Moreover, as in our cases, patients usually undergo total body imaging to underline further distant site of metastatic disease. After complete patient staging, multidisciplinary approach should be utilized for every patient to individualize care.

Optimal management of BC_iv_ with BM remains controversial. Surgery, WBRT, stereotaxis radiosurgery (SRS) and chemotherapy are considered the cornerstones of treatment for BM in addition to corticosteroids for symptom-based therapy. In contrast with lung or hepatic metastasis treatment guidelines, evidence in BC_iv_ with BM is missing and only few studies suggest a survival benefit in patients who associate WBRT with PRT [17]. However, surgical resection followed by radiotherapy is the gold standard of treatment in patient with single site BM as in our patient [18]. As mentioned before, role of breast surgery in *de novo* BCiv patients is still unclear and this role is decreased in the clinical practice in the last 30 years. PTR occurs in nearly half of the cases [4]. Despite the reduction of surgery, when mastectomy is performed, BC_iv_ patients reconstruction rate increased from 5.2% in 2004 to 14.3% in 2013 [5]. Three different metanalysis from literature demonstrated higher survivorship rate of patients when PTR is performed with better distant progression free survival, but no improvement in progression free survival [[6], [7], [8]]. Moreover, in the larger and more recent metanalysis by Xiao *et al.,* PTR seems to improve survivorship in oligometastatic disease BC_iv_ patient (one metastatic site, bone only metastatic disease, surgery with negative margins HR = 0.62, P < 0.001; HR = 0.61, P = 0.05; HR = 0.61; P < 0.001 respectively). Nevertheless, retrospective design, selection *bias* and heterogeneity of population among study could overestimate the role of PTR in BC_iv_ patient, confirming the need of further study and careful selection of patients to address to surgery [8].

In our clinical practice we report a successful combined surgical approach in stage IV de novo BC patients with single site BM at one year of follow up. In our clinical case, metastasectomy plus mastectomy provided neurological control of acute complication of metastatic disease and complete BC local control. Immediate two-time breast reconstruction gave us the possibility to start the correct reconstructive protocol [19]. One-time operation could be the best option when diagnosis of breast cancer is made thanks to the onset of oncological emergency like intracranial hypertension due to single brain metastasis. Moreover, combined surgical approach provides less impact of anesthesiologic and surgical stress on immunological response [20,21].

## 4 Conclusion

Systemic medical therapy has improved the survival of patients at stage 4 de novo of breast cancer and evidence of literature in metanalysis from large retrospective cohort demonstrated how primary tumor surgery seems to improve patients’ prognosis under particular clinical condition.

In our opinion, despite prognosis remains poor in stage IV de novo patients, risks and benefits of breast reconstruction should always be discussed with patients when mastectomy is performed, in order to provide an improvement of quality of life in long survivors.

Combined surgical approach offers the opportunity to treat two different oncological urgencies, reducing the unnecessary repeated surgical and anesthesiologic trauma.

## Declaration of Competing Interest

We declare no conflict of interest.

## Sources of funding

Nothing to declare.

## Ethical approval

For this study, ethical and ethnical approval are not required.

## Consent

Written informed consent was obtained from the patient for publication of this case report and accompanying images. A copy of written consent is available for review by the Editor-in-Chief of this journal on request.

## Author contribution

**Francesca Santori:** Conceptualization, Data curation, Formal analysis, Writing - original draft, Writing - review & editing. **Gianluca Vanni:** Conceptualization, Data curation, Formal analysis, Writing - review & editing. **Oreste Claudio Buonomo:** Conceptualization, Data curation, Formal analysis, Writing - review & editing. **Adriano De Majo:** Data curation, Formal analysis, Writing - review & editing. **Maurizio Rho:** Data curation, Formal analysis, Writing - review & editing. **Alessandra Vittoria Granai:** Data curation, Formal analysis, Writing - review & editing. **Marco Pellicciaro:** Data curation, Formal analysis, Writing - review & editing. **Maria Cotesta:** Data curation, Formal analysis, Writing - review & editing. **Massimo Assogna:** Data curation, Formal analysis, Writing - review & editing. **Rolando Maria D’Angelillo:** Data curation, Formal analysis, Writing - review & editing. **Marco Materazzo:** Conceptualization, Data curation, Formal analysis, Writing - review & editing.

## Registration of research studies

Case report not registered.

## Guarantor

Francesca Santori and Marco Materazzo.

## Provenance and peer review

Not commissioned, externally peer-reviewed.
